# Clinical Relapses of Atypical HUS on Eculizumab: Clinical Gap for Monitoring and Individualised Therapy

**DOI:** 10.1155/2018/2781789

**Published:** 2018-02-06

**Authors:** Chia Wei Teoh, Kathleen Mary Gorman, Bryan Lynch, Timothy H. J. Goodship, Niamh Marie Dolan, Mary Waldron, Michael Riordan, Atif Awan

**Affiliations:** ^1^Division of Nephrology, The Hospital for Sick Children, Toronto, ON, Canada; ^2^Department of Paediatric Nephrology & Transplantation, The Children's University Hospital, Temple Street, Dublin 1, Ireland; ^3^Department of Paediatrics, University of Toronto, Toronto, ON, Canada; ^4^Department of Neurology, The Children's University Hospital, Temple Street, Dublin 1, Ireland; ^5^Institute of Genetic Medicine, Newcastle University, International Centre for Life, Central Parkway, Newcastle upon Tyne, UK; ^6^Department of Paediatric Nephrology, Our Lady's Children's Hospital, Crumlin, Dublin 12., Ireland

## Abstract

Atypical hemolytic uremic syndrome (aHUS) is caused by dysregulation of the complement system. A humanised anti-C5 monoclonal antibody (eculizumab) is available for the treatment of aHUS. We present the first description of atypical HUS in a child with a coexistent diagnosis of a POL-III leukodystrophy. On standard eculizumab dosing regime, there was evidence of ongoing C5 cleavage and clinical relapses when immunologically challenged. Eculizumab is an effective therapy for aHUS, but the recommended doses may not be adequate for all patients, highlighting the need for ongoing efforts to develop a strategy for monitoring of treatment efficacy and potential individualisation of therapy.

## 1. Introduction

Atypical hemolytic uremic syndrome (aHUS) is a rare disorder characterised by thrombotic microangiopathy and renal failure. It is a disease of complement dysregulation associated with poor prognosis and high mortality with up to 50% progressing to end-stage renal disease [1]. It accounts for 5–10% of children presenting with HUS and approximately 60% of cases are associated with an inherited and/or acquired abnormality of complement [2].

In 2011, a humanised anti-C5 monoclonal antibody (eculizumab) was licenced for treating aHUS in the USA and Europe. The efficacy and safety of its use were demonstrated in adults and children with aHUS, with and without identified pathogenic variants in complement activating/regulating factors or CFH-autoantibodies [3–6]. Eculizumab therapy in aHUS caused by a gain-of-function pathogenic variant of complement factor B* (CFB)* is described [7]. Despite excellent clinical response with standard recommended dosage, there was laboratory evidence of ongoing excessive complement activation.

POL-III [4H leukodystrophy (4H)] leukodystrophy is characterised by a triad of hypomyelination, hypodontia, and hypogonadotropic hypogonadism [8]. First described in 2003, it consists of 5 overlapping phenotypes, which were initially presented as similar but distinct disease entities [8]. Pathogenic variants in 2 genes coding for subunits of RNA polymerase III,* POLR3a* (10q22.3) and* POLR3b* (12q23.3), were identified with genotype-phenotype correlation. Variants in* POL3a* are more severely affected with a later onset, faster regression, and shorter life [9].

We provide the first report of aHUS in a patient with POL-III leukodystrophy. There were clinical relapses when immunologically challenged and laboratory evidence of excessive complement activation on eculizumab therapy. This case emphasises the need to develop a strategy for monitoring treatment efficacy and potential individualisation of anticomplement therapy. It also highlights the need to consider alternative diagnoses should there be discordant genotype-phenotype features.

## 2. Case Report

A previously well male infant born to nonconsanguineous Irish parents presented at 9 months old with a two-day history of fever, vomiting, nonbloody diarrhea, irritability, and poor feeding. This was preceded by an upper respiratory tract infection (URTI) a week prior to presentation. He received a second dose of H1N1 vaccine two weeks previously. At presentation, he was pale and irritable with petechial rash, had microscopic hematuria and proteinuria, and was hypertensive (systolic blood pressure of 140 mmHg). He developed hypertensive encephalopathy with seizures requiring labetalol, sodium nitroprusside, and phenytoin infusions.

Investigations at presentation revealed thrombotic microangiopathy (hemoglobin, 105 g/L; platelets 77 × 10^9^/L; lactate dehydrogenase (LDH) 4795 U/L, blood film showed numerous red cell fragments) and impaired renal function (urea 8.5 mmol/L, creatinine 90 *μ*mol/L). Stools were positive for rotavirus but negative for* E. coli* 0157 and verotoxin. Urinary protein/creatinine ratio (PCR) was 2851 mg/mmol. The patient's ADAMTS13 activity, complement C3 and C4 were normal. A diagnosis of aHUS was suspected, direct sequencing of the entire coding regions for* CFH*,* CFI*,* CD46*,* DGKε*, and* C3* did not reveal any disease causing variant and multiplex ligation-dependant probe amplification analysis for deletions, and duplications of* CFH*,* CFHR1*, and* CFHR3* exons were negative. Sequencing of* CFB* identified a missense heterozygous variant of c.724A>C (Chr 6:31915584 (p.lle242Leu, rs144812066)), a benign variant as it was identified in his phenotypically normal mother, common in ExAC control database and predicted to be benign by in silico programs.

He received eight daily sessions of plasma exchanges (PE) with clinical improvement and subsequently relapsed on day 15 leading to resumption of daily PE. He required at least twice weekly PE but was gradually weaned over the course of 6 months, until eventual discontinuation at 15 months old (total 64 sessions of PE). Although LDH remained persistently elevated (620–925 U/L), full blood count (FBC), plasma creatinine, and haptoglobin levels normalised. Urinary PCR fell to <50 mg/mmol. He remained in clinical remission for 18 months.

**Figure 1:**
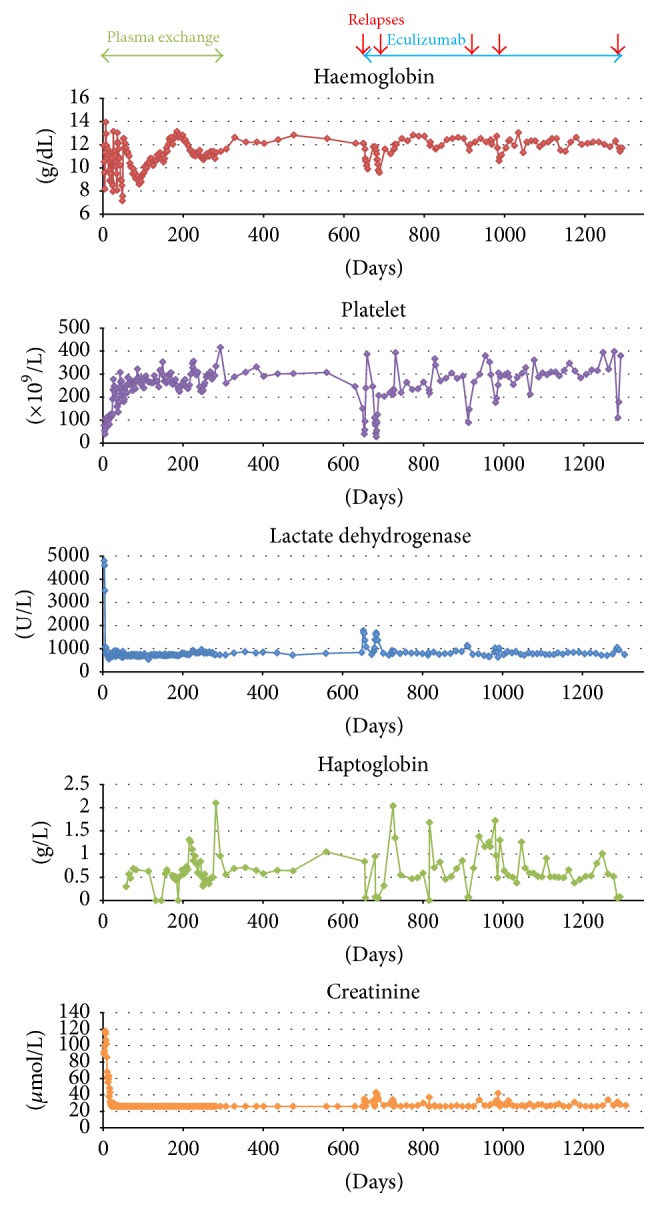
Graphical illustration of aHUS patient's clinical course and treatment. Clinical relapses depicted by red arrows.

The first clinical relapse of aHUS occurred at 33 months of age, preceded by an URTI (hemoglobin 102 g/L, platelets 39 × 10^9^/L, LDH 1785 U/L, haptoglobin 0.06 g/L, and creatinine 35 *μ*mol/L). He received the first dose of eculizumab 600 mg, which led to clinical, haematological, and biochemical improvement within two days, and was maintained on fortnightly doses (300 mg) thereafter. On fortnightly eculizumab regimen, he presented with three relapses, in the setting of immunological challenges (vaccination and URTI). Clinical, haematological, and biochemical improvement occurred within 2-3 days of eculizumab infusion with each relapse (Figure 1).

Since the first relapse, he remained hypertensive requiring captopril, amlodipine, and carvedilol. On fortnightly eculizumab, FBC, plasma creatinine, haptoglobin, and C3/C4 levels remained within normal limits, with persistently elevated LDH (630–881 U/L). The alternative and classical complement pathways were suppressed confirming drug action (CH50 and AP50 hemolytic assays < 10%). Although clinically in remission (Hb 122 g/L, platelets 380 × 10^9^/L, LDH 707 U/L, haptoglobin 1.16 g/L, and creatinine 26 *μ*mol/L), plasma sC5b-9 concentrations measured 14 days after eculizumab dose were high at 240 ng/ml. The high sC5b-9 was interpreted as ongoing dysregulated complement activation and his maintenance eculizumab dose was changed to 300 mg every 10 days. Eight weeks after decreasing the interdose interval, sC5b-9 concentration remained elevated at 227 ng/ml (10 days after eculizumab dose) despite clinical remission (Hb 125 g/L, platelet 309 × 10^9^/L, LDH 756 U/L, haptoglobin 0.51 g/L, and creatinine 27 *μ*mol/L) and adequate trough eculizumab levels (>600 *μ*g/ml) with levels >99 *μ*g/ml considered to be therapeutic.

During follow-up, it became evident that he had global developmental delay. Careful review of his history demonstrated subtle gross and fine motor delays were present prior to his initial presentation with aHUS (sat with support at 9 months, not reaching for objects/transferring). He made slow developmental progress, without regression. At 4 years, he could stand with support, had palmar but no pincer grip, and only two words with meaning. He had good understanding, could follow 4-step commands, and was sociable and interactive. Examination at 4 years showed occipitofrontal circumference less than the 0.2nd centile, bilateral optic nerve pallor, hypodontia, truncal ataxia, upper limb tremor, and hypotonia. Therefore, an alternate pathology to the aHUS and associated hypertensive encephalopathy was required to explain the preexisting developmental delay and neurological deficits.

A MRI brain performed at 10 months following initial presentation showed subtle changes in the basal ganglia. He underwent extensive genetic and metabolic work-up including karyotype, microarray, PLP gene, very long chain fatty acids, paired serum and CSF glucose and amino acids, CSF neurotransmitters, urinary organic acids, and skin and muscle biopsy, all of which were negative. Two subsequent MRI brain scans at 2.25 and 3.5 years showed very delayed myelination with no progression since the original scan showing hypomyelination, increased T2 signal in the basal ganglia with volume loss, and cerebellar atrophy. Based upon these findings, he was diagnosed with 4H syndrome with subsequent confirmation of compound heterozygous mutation of* POL3B* (c.1568T>A, p. Val523Glu and c. 1464+1 G>A), reported previously [10].

Eculizumab treatment was discontinued after careful consideration with his parents due to ongoing relapses and the diagnosis of a progressive severe neurological condition.

## 3. Discussion

Pathogenic variants in* CFB* result in the formation of C3bBb (C3 convertase) which is resistant to decay by CFH and decay accelerating factor, resulting in increased quantities of active convertase [7]. Eculizumab is an anti-C5 monoclonal antibody, which prevents cleavage of C5 by C5 convertase, preventing the assembly of the membrane attack complex and generation of C5a. It is effective in treating aHUS associated with a variety of complement disorders [3–6, 11–13], including CFB mutation [7].

In our patient, a persistent, mildly elevated LDH and clinical relapses while on eculizumab prompted further investigations which showed that although the classical and alternative pathways were fully suppressed (CH50 and AP50 levels < 10%), plasma sC5b-9 concentration remained elevated, a finding which persisted despite reducing the interdose interval to 10 days, with high trough drug levels. This finding has been previously reported [14, 15], suggesting ongoing C5 cleavage that may explain the relapses. Volokhina et al. reported normalisation of sC5b-9 levels during remission [16]. The use of soluble complement profile as diagnostic and/or prognostic biomarkers remains debatable as highlighted by Noris et al. who reported inconsistent complement profiles in patients with aHUS regardless of whether they had any complement genetic mutations [17]. In that study, up to 64% of patients had markers of complement activation which persisted in a subgroup of patients despite clinical remission with treatment [17]. These discrepancies emphasise the need to develop a strategy for monitoring treatment efficacy that can be used to individualise eculizumab therapy in patients with aHUS [18].

The heterozygous variant in CFB did not explain our patient's phenotype. Whole exome sequencing did not identify any other disease causing variant. Up to 40% of patients with aHUS have no identified disease-causing variant [2]. Our patient also had a clinical and genetically confirmed diagnosis of 4H leukodystrophy. To date, there are no reports of any association with aHUS or defects in the complement system. 4H leukodystrophy is a spectrum of disorders with genotype-phenotype correlations [9]. The variant in our patient has been described previously but no further information is available on his clinical course [10]. This case emphasises the importance of considering separate diagnoses in a patient with a complex medical history. The clinical picture in this setting could have been assumed to be due to his prolonged illness and ICU admission at presentation. However, identification of preceding neurodevelopmental delay, clinical examinations, and recognition of typical MRI features of 4H leukodystrophy were key to the diagnosis [9].

## 4. Conclusion

We report an aHUS patient with no identified pathogenic variant, who responded to eculizumab therapy, but with ongoing clinical relapses during periods of immunological challenges, despite adequate drug levels. To our knowledge, he is the only patient with aHUS to be diagnosed with 4H leukodystrophy, which became apparent on follow-up with neurodevelopmental delay that was not in keeping with the initial diagnosis. Our case highlights the importance of maintaining a high index of suspicion for a separate diagnosis in a patient with a complex medical history, especially with discordant genotype-phenotype features. Further studies are necessary to reconcile these conflicting results (absent CH50/AP50 activity with elevated sC5b-9 concentration), to ascertain the best method for monitoring adequacy of eculizumab therapy to inform future individualisation of aHUS therapy.

## References

[1] Noris M., Remuzzi G. (2009). Atypical hemolytic-uremic syndrome. *The New England Journal of Medicine*.

[2] Loirat C., Frémeaux-Bacchi V. (2011). Atypical hemolytic uremic syndrome. *Orphanet Journal of Rare Diseases*.

[3] Legendre C. M., Licht C., Muus P. (2013). Terminal complement inhibitor eculizumab in atypical hemolytic-uremic syndrome. *The New England Journal of Medicine*.

[4] Licht C., Greenbaum L. A., Muus P. (2015). Efficacy and safety of eculizumab in atypical hemolytic uremic syndrome from 2-year extensions of phase 2 studies. *Kidney International*.

[5] Greenbaum L. A., Fila M., Ardissino G. (2016). Eculizumab is a safe and effective treatment in pediatric patients with atypical hemolytic uremic syndrome. *Kidney International*.

[6] Fakhouri F., Hourmant M., Campistol J. M. (2016). Terminal complement inhibitor eculizumab in adult patients with atypical hemolytic uremic syndrome: a single-arm, open-label trial. *American Journal of Kidney Diseases*.

[7] Gilbert R. D., Fowler D. J., Angus E., Hardy S. A., Stanley L., Goodship T. H. (2013). Eculizumab therapy for atypical haemolytic uraemic syndrome due to a gain-of-function mutation of complement factor B. *Pediatric Nephrology*.

[8] Atrouni S., Darazé A., Tamraz J., Cassia A., Caillaud C., Mégarbané A. (2003). Leukodystrophy associated with oligodontia in a a large inbred family: fortuitous association or new entity?. *American Journal of Medical Genetics*.

[9] Wolf N. I., Vanderver A., van Spaendonk R. M. (2014). Clinical spectrum of 4H leukodystrophy caused by *POLR3A* and *POLR3B* mutations. *Neurology*.

[10] Tétreault M., Choquet K., Orcesi S. (2011). Recessive mutations in *POLR3B*, encoding the second largest subunit of Pol III, cause a rare hypomyelinating leukodystrophy. *American Journal of Human Genetics*.

[11] Besbas N., Gulhan B., Karpman D. (2013). Neonatal onset atypical hemolytic uremic syndrome successfully treated with eculizumab. *Pediatric Nephrology*.

[12] Cayci F. S., Cakar N., Hancer V. S., Uncu N., Acar B., Gur G. (2012). Eculizumab therapy in a child with hemolytic uremic syndrome and CFI mutation. *Pediatric Nephrology*.

[13] Al-Akash S. I., Almond P. S., Savell V. H., Gharaybeh S. I., Hogue C. (2011). Eculizumab induces long-term remission in recurrent post-transplant HUS associated with C3 gene mutation. *Pediatric Nephrology*.

[14] Goicoechea de Jorge E., Harris C. L., Esparza-Gordillo J. (2007). Gain-of-function mutations in complement factor B are associated with atypical hemolytic uremic syndrome. *Proceedings of the National Acadamy of Sciences of the United States of America*.

[15] Vilalta R., Lara E., Madrid A. (2012). Long-term eculizumab improves clinical outcomes in atypical hemolytic uremic syndrome. *Pediatric Nephrology*.

[16] Volokhina E. B., Westra D., van der Velden T. J. A. M., van de Kar N. C. A. J., Mollnes T. E., van den Heuvel L. P. (2015). Complement activation patterns in atypical haemolytic uraemic syndrome during acute phase and in remission. *Clinical & Experimental Immunology*.

[17] Noris M., Galbusera M., Gastoldi S. (2014). Dynamics of complement activation in aHUS and how to monitor eculizumab therapy. *Blood*.

[18] Teoh C. W., Riedl M., Licht C. (2016). The alternative pathway of complement and the thrombotic microangiopathies. *Transfusion and Apheresis Science*.

